# Personalized Treatment Decisions for Traumatic Proximal Finger Amputations: A Retrospective Cohort Study

**DOI:** 10.3390/jpm13020215

**Published:** 2023-01-26

**Authors:** Nadjib Dastagir, Doha Obed, Khaled Dastagir, Peter M. Vogt

**Affiliations:** Department of Plastic, Aesthetic, Hand and Reconstructive Surgery, Hannover Medical School, 30625 Hannover, Germany

**Keywords:** amputation, hand injury, plastic and hand surgery, wound healing

## Abstract

Ray and proximal phalanx amputations present valid surgical options for the management of severe traumatic finger injuries. However, among these procedures, the superior one for optimal functionality and quality of life for patients still remains unknown. This retrospective cohort study compares the postoperative effects of each amputation type to provide objective evidence and to create a paradigm for clinical decision-making. A total of forty patients who had received either ray or proximal phalanx-level amputations reported on their functional outcomes using a combination of questionnaires and clinical testing. We found a decreased overall DASH score following ray amputation. Particularly, Part A and Part C of the DASH questionnaire were consistently lower compared with amputation at the proximal phalanx. Pain measurements in the affected hand were also significantly decreased during work and at rest in ray amputation patients, and they reported decreased cold sensitivity. Range of motion and grip strength were lower in ray amputations, which is an important preoperative consideration. We found no significant differences in reported health condition, evaluated according to the EQ-5D-5L, and blood circulation in the affected hand. We present an algorithm for clinical decision-making based on patients’ preferences to personalize treatment.

## 1. Introduction

Finger amputations often result from devastating hand injuries, with an estimated 45,000 operations performed per year in the US [1,2]. Amputations are commonly due to accidental traumatic injury, congenital deformity, or acquired pathological conditions. Treatment of traumatic injuries requires immediate intervention, whereas congenital deformities need elective surgery. Additional causes for elective surgeries include the management of chronic conditions, such as peripheral arterial disease (PAD) and Dupuytren’s contracture, or cancer resection [3,4,5]. The highest incidence rates of finger amputations are found in children younger than five years and adults over sixty-five years [2]. A decrease in finger replantation surgeries has been reported in the US, while cases of finger amputations have remained the same [6]. This suggests that finger amputation remains a prominent treatment strategy for severe finger and hand injuries in many clinics.

Fingers are an important organ for prehension, communication, and sensation. Finger amputations can have detrimental functional and psychological effects, leading to drastic changes in quality of life [7]. Hands are not only important for fine and gross motor skills, but also serve a vital sensory role. As one of the most innervated regions of skin on the body, the fingers‘ sensory system comprises many delicate receptors that absorb a wide range of environmental inputs and stimuli [8]. These thousands of nerve endings in the fingertips allow the body to process and recognize heat, touch, and pain. Fingers also serve as communication tools frequently used for gesticulation and self-expression [9,10]. In addition to the functional losses of the hand following finger amputation, patients often deal with ongoing psychological effects. One study examining the role of finger amputation reported that 96% (*n* = 25) of affected patients reported symptoms such as anxiety, depression, or post-traumatic stress disorder (PTSD) in the 3 months following their amputation [11]. Such psychological effects can decrease the quality of life for patients. Based on the hand’s roles in motor function, sensation, and psychology, it is important that clinicians understand how finger amputation affects patients in their daily lives.

With advances in surgical techniques and an increase in documentation of postoperative outcomes, clinicians and patients are more frequently able to make evidence-based surgical decisions. When patients present with severe traumatic injuries to the finger between the proximal interphalangeal (PIP) and metacarpal phalangeal (MCP) joints, e.g., as the result of an accidental traumatic injury, they can decide to undergo ray amputation or proximal phalanx amputation, which leaves a residual stump. The literature is conflicting with regard to the superiority and advantages and disadvantages of these procedures.

Ray amputation is appropriate when there is loss of the proximal phalangeal skeleton or PIP joint function [7,12]. While ray amputation can be more disabling by reducing grip strength, it minimizes “gapping” of the hand, which might otherwise result from a remnant digit stump. This may be preferred by patients, as the cosmetic outcome minimizes the absence of the digit [13,14]. Depending on the amputated finger, with the exclusion of the thumb, the rest of the digits can adapt, resulting in a functional hand. Patients who decide to undergo proximal phalanx amputation are frequently concerned about the loss of strength due to the consequential narrowing of the hand in ray amputation [15,16]. There is the possibility of obstruction from the remnant partial digit and stiffness resulting in loss of function and dexterity, which can ultimately decrease hand function [12,17].

There are conflicting reports regarding subsequent functional strength and quality of life following these two amputation methods [18,19,20,21,22]. Ray amputation has been shown to significantly decrease grip and pinch strength compared with patients’ unaffected hands, which can be an important consideration for patients deciding on the amputation level, particularly if they require the use of their hands in their work [7,19]. There is also concern about phantom pain symptoms, which can occur from the formation of a neuroma [20]. The remnant finger stump following proximal phalanx amputation can be an important consideration as phantom pain can be more severe, as the nerve ending is close to the end of the stump and can be regularly traumatized [21]. Ray amputation patients reported less pain but also decreased sensitivity in their affected hand in [18]. Both surgery types show comparable postoperative returns to work, being around two months [19]. Postoperative cosmesis can be important to patients, with some preferring proximal phalanx amputation, as they retain a normal finger count and can use a prosthetic over the stump, and others prefer ray amputation, as the absence of the digit is less noticeable. Due to the different nature of these two amputations, it is vital that patients and clinicians choose the surgery that is personalized to each patient’s needs.

Therefore, it is important to understand how different amputation levels contribute to functional outcomes of the surgical procedure [22,23]. Patients will have different preferences depending on their profession, lifestyle, and cosmetic concerns. The use of patient-reported outcome (PRO) measures is important to allow clinicians and patients to make informed preoperative decisions with the intent to maximize postoperative satisfaction.

This retrospective cohort study aims to answer which type of amputation, ray or proximal phalanx, results in the highest functionality and quality of life for patients. By comparing cohorts of each amputation model using PROs, strength tests, and blood flow quantification, we provide an algorithm that can serve as a guide for choosing the surgical technique based on a patient’s preferences to increase the personalization of treatment.

## 2. Materials and Methods

A total of 40 patients who received amputation of a single finger between 2017 and 2022 were examined in this retrospective cohort study. Two cohorts of patients who underwent finger amputation were recruited. Patients were study-eligible if they received either ray amputation or amputation at the proximal phalanx of one finger. Patient eligibility criteria included being at least 18 years old, having a good understanding of the German language, and documented follow-up at least 12 months after amputation. The study was performed in accordance with the ethical standards of the Declaration of Helsinki and was approved by the local ethical review committee [No. 7887_BO_K_2018]. All patients voluntarily participated in the present study. Prior to participation, a consent form was signed and personally dated by patients and surgeons. The decision-making for the amputation type was based on the surgeon’s experience, the characteristics of the injury, and discussion with the patient.

The subjective outcomes and manual capability of the affected hands were evaluated using the Disabilities of the Arm, Shoulder, and Hand (DASH) questionnaire. Patients were asked to rank functionality (Part A), pain (Part B), and sports and music (Part C) on 3 levels using a scale from 0 to 100 indicating minimum–maximum disability. The impact of amputations on wrists was evaluated using Patient-Rated Wrist Evaluation (PRWE). This questionnaire consisted of 2 parts with the first evaluating wrist pain and the second wrist function ranked on a 0 to 100 scale, with 0 representing no restriction and 100 maximum disability.

This study characterized hand functionality as the differences in gross strength, sensibility, pain perception, range of motion, and blood circulation between the patients’ postoperative and unaffected hands. Force measurements were made using a JAMAR dynamometer and pinch meter. Assessment of sensibility was conducted using two-point discrimination (2PD). Range of motion was measured via the neutral-zero method. Blood circulation efficiency in the affected hands was measured using a forward-looking infrared (FLIR) thermal imaging camera and laser speckle contrast analysis (LASCA).

The general quality of life and condition of the patients was assessed using the European Quality of Life 5 Dimensions 5 Level Version (EQ-5D-5L). This PRO system uses six questions as well as a vertical EuroQol visual analog scale (EQ-VAS). The EQ-VAS score is a hybrid of VAS and a hash-marked numerical rating scale, with 0 representing the lowest and 100 the highest health profile.

Cold sensitivity was assessed by asking patients if exposure to cold air or cold water provoked sensitivity in their affected fingers. To assess cosmesis, patients ranked their aesthetic satisfaction with the amputation on a scale of “Excellent”, “Good”, or “Unsatisfactory”.

### Statistics

The sample size was determined as a minimum of 20 patients per group for Fisher’s exact test using Statistical Power Analyses Software, G*Power (Düsseldorf, Germany).

Comparisons between the two groups were analyzed for statistical significance using a paired *t*-test. Two-way ANOVAs were used for comparisons between more than two groups. All statistical analyses were performed using GraphPad Prism 9.2.0. Differences were considered significant if *p* < 0.05.

## 3. Results

### 3.1. Patient Demographics

Of the 40 patients, 50% had received ray amputations. The mean age at the time of operation was 47.5 years, with ray amputation patients averaging 49.5 years and proximal phalanx amputation patients averaging 45.5 years (Table 1). Forty-three percent of the patients were female, constituting sixty percent of the ray amputation patients and twenty-five percent of the proximal phalanx amputation patients. The mean time of examination post-trauma was 3.5 years (with a range of 3–4 years) for the patients with proximal phalangeal finger amputations and 4.3 years for the patients with ray amputations (with a range of 2–5 years). The majority of amputations occurred on the non-dominant hand for both amputation types. The amputated finger on the hand ranged from digitus 2 to digitus 5. The highest amputation prevalence occurred on digitus 2 followed by digitus 4 for both amputation treatments. While the cause of injury varied, 30% of patients reported their injury being caused by a chainsaw accident. To further understand the advantages and disadvantages of these two operation types, we subdivided our cohorts based on employment, sex, and affected hand dominance.

### 3.2. DASH Scores Indicate Higher Reported Quality of Life Following Ray Amputations

To determine patients’ postoperative quality of life we used the DASH scoring system. Patients who received ray amputation had a significantly lower overall DASH score of 33.88 compared with the proximal phalanx patients with a mean score of 50.13 (Table 2). Assessment of wrist pain and function using the PRWE questionnaire also showed significantly increased disability following proximal phalanx amputation. To measure patient healthiness, the EQ-5D-5L questionnaire was used. There were no significant differences between cohorts. EQ-VAS scoring showed significantly higher scores for ray resectioning with a mean score of 82.89 compared with mid-phalanx resection patients with a mean score of 72.37.

Further subclassification of the patient groups showed significantly lower DASH functionality scores (Part A of the questionnaire) in manual workers, females and males, and dominant-hand-affected patients following ray amputation in comparison with the proximal phalanx level (Table 3). A similar trend was seen in Part C of the DASH questionnaire, pertaining to sports and music, where ray amputation resulted in a significantly lower mean score in manual workers and females, and was decreased in non-manual workers and non-dominant-hand-affected patients. Overall, ray amputation showed lower mean scores compared with proximal phalanx amputation with the exception of Part B which assesses work and pain symptoms.

### 3.3. Diverse Functional Outcomes Following Ray Amputation

Following amputation, it is important that patients comprehend the effects of the surgical procedure on overall hand strength and range of motion. Patients who underwent ray amputation had 45% less stationary pain in the area of the operated hand and 33% less working pain compared with patients with a residual stump (Table 4). Grip strength in patients who received ray amputation was 20% lower compared with those who received proximal phalanx amputation. Range of motion was also decreased in 15% of ray amputation patients compared with proximal phalanx amputees who retained 100% compared with their unaffected hands. Additionally, we observed reduced cold sensitivity following ray amputation occurring in 42% of patients compared with 75% of proximal phalanx patients.

### 3.4. Higher Cosmesis Following Ray Amputation

Sixty-five percent of ray amputation patients rated the cosmetic result as excellent, compared with 30 percent of the proximal phalanx amputation patients (Table 4). Patients who received proximal phalanx amputation were more likely to rank the aesthetic results as good, with 60% agreeing, compared with 32.50% of ray amputation patients. Of proximal phalanx patients, 10% were dissatisfied with the cosmetic outcome compared with just 2.5% of ray patients.

### 3.5. No Significant Changes in the Affected Hands’ Circulation

**Figure 1:**
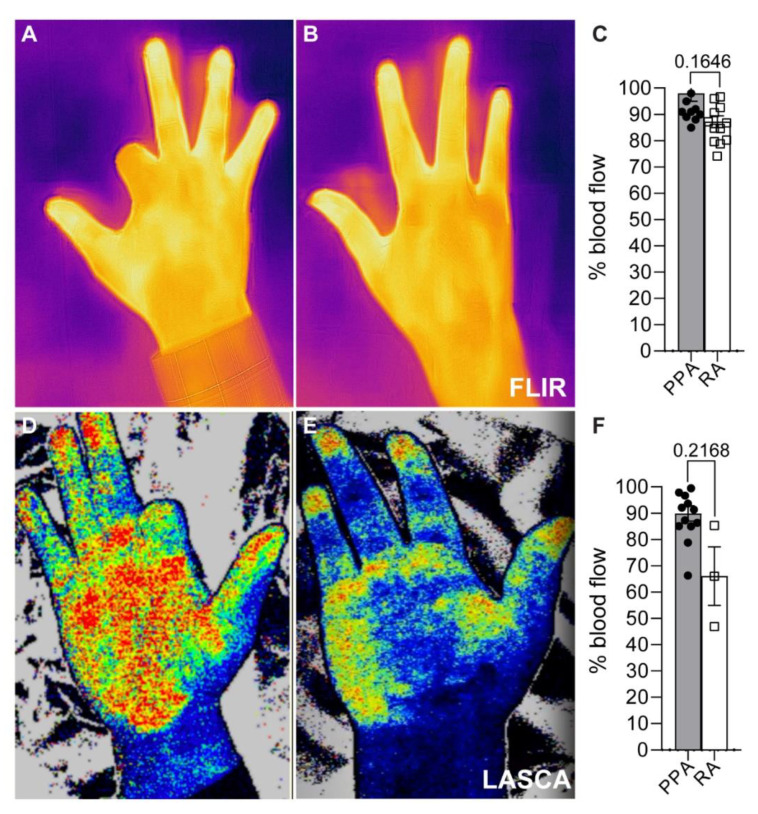
FLIR and LASCA blood circulation measurements in affected hands. (**A**–**C**) Representative images and quantification of FLIR. (**D**–**F**) Representative LASCA images and quantification. Percentage blood flow assessed in comparison with the unaffected hands. Results are means ± SEM.

Resumption of adequate blood flow is essential to promote proper healing post-amputation. Hence, we measured blood flow using FLIR and LASCA. There were no significant differences in skin temperature and blood flow in the affected hands between the two groups (Figure 1).

### 3.6. Flow Chart for Clinical Decision-Making between Ray and Proximal Phalanx Amputations

**Figure 2:**
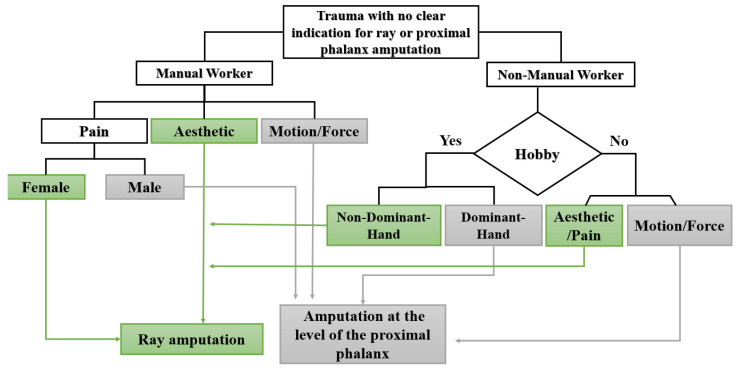
Algorithm for ray vs proximal phalanx amputation based on patients’ preferences. Hobby refers to recreational activities that require extended use of the hand, such as playing a musical instrument or sports.

Based on our analyses of patient quality of life, functionality, and postoperative pain, we present a stratified flow chart to enable personalized patient decision-making regarding ray and proximal phalanx level amputations (Figure 2). Indicative factors for ray amputation are shown in green, while gray indicates factors that may lead to a preference for proximal phalanx amputation. Notably, patients concerned with aesthetics, postoperative pain, and hand dominance are shown to prefer the outcomes of ray amputation. In contrast, manual workers and non-manual workers with hobbies such as sports and music, who are concerned about the motion and strength of their affected hands, are more likely to favor amputation at the level of the proximal phalanx.

## 4. Discussion

In this retrospective cohort study, we compared the two most common finger amputation methods: ray amputation and proximal phalanx amputation. Using PRO measures, and stratification based on patient demographics, we identified factors that predict the suitability of ray versus proximal phalanx amputation based on a patient’s needs and concerns. We found notable differences between the choices of amputation type based on employment status, pain, aesthetics, and hobbies. Our results indicate patients concerned about aesthetics and pain management prefer the outcomes of ray amputation, while patients concerned about strength and motion prefer proximal phalanx amputation. The overall DASH score of ray amputation patients was 33% lower compared with that of proximal phalanx patients, the mean PRWE was 58% lower, and EQ-VAS scoring was 13% higher, all suggesting higher functional outcomes of ray amputation. The stratification of DASH scores based on employment type, sex, and hand dominance showed mean ray resection scores were lower compared with those of proximal phalanx amputation with the exception of Part B, pain and symptoms. We observed significantly lower scores in Part A, functionality, in both females and males, and in patients who indicated their affected hand as dominant. Part B showed comparable scores between ray and proximal phalanx amputation, with no significant differences. The results from Part C indicate a preference for ray amputation in manual workers and females. Prior to surgery, when there is an option to decide on the amputation level, it is important to consider the patient’s needs and preferences to allow for the personalization of treatment and to maximize postoperative satisfaction.

Ray amputation was preferable in cases where patients were primarily concerned about pain management and aesthetic preferences and if their non-dominant hand was affected. Interestingly, there was a sex difference between pain management preferences, with females reporting decreased pain following ray amputation. This is likely to be because females tend to experience increased phantom limb pain, which is more common in proximal phalanx level amputation [24]. Additionally, ray amputation showed significantly reduced working and resting pain levels based on numeric pain rating measurement, as well as reduced cold sensitivity, which has been previously reported in [25]. These results are similar to a study by Karle et al., which found reduced cold and weather sensitivity in 55.2% of ray amputation patients compared with 33.3% of proximal phalanx amputation patients [18]. Overall healthiness, as assessed by the EQ-VAS questionnaire, showed significantly higher scoring in ray amputation patients. These pain and sensitivity considerations might be useful for patients who are concerned about post-amputation daily life and well-being.

Our results found ray amputation to be cosmetically more desirable. This is in line with previous reports, highlighting that ray amputations deliver aesthetically more favorable results, particularly when the middle and ring fingers are affected [26]. Ray amputation minimizes the noticeability of the missing finger by removing any stump and forming a connecting web [27,28]. Cultural preferences can also play a role when patients decide if they want to undergo ray amputation or leave a residual finger stump [29]. Of the patients participating in this study, those with ray amputations showed the highest cosmesis; however, this is highly dependent on patients’ subjective opinions.

Dominant hand preference is also useful to consider when deciding on the type of amputation. In this study, a patient’s dominant hand was defined as the preferred hand for writing and most frequently used in daily activities. Hand dominance is often associated with fine motor skills, with the non-dominant hands demonstrating reduced coordination and speed [30]. Ray amputation was shown to be preferable in Part C, on recreational activities, of the DASH scoring when the non-dominant hand was affected. This preference might be because the operation minimizes the gapping between fingers by closing the interdigital space and results in a more symmetrical hand. This enhances the use of the hand as an entity and can make the absence of the digit less noticeable. As previously reported, the remaining digits on the hand quickly adapt, which can improve overall functionality [25]. This can be useful if the patient engages in recreational sports or music activities that require the use of their non-dominant hand. Ray amputation has the potential for complications as a result of the narrowing of the hand and the technical challenges of the surgery. When the metacarpal gap is not closed correctly, this can result in issues such as scissoring and malalignment. When this occurs, the hand function may decrease as well as the overall cosmesis [7,31]. When determining which amputation type is most suitable for a patient, it is useful to consider hand dominance. In the case of our study, ray amputation was preferred for non-dominant hand injuries, particularly in patients that frequently use their hands in recreational activities.

Proximal phalanx amputation was preferable in cases where the patient was primarily concerned with the motion and force of the hand or where their dominant hand was affected. Motion and force of the hand tend to be more important for manual workers who might otherwise not be able to continue their careers. In cases where employment is closely tied to hand dexterity, amputation at the level of the proximal phalanx is ideal. Patients with hobbies involving their dominant hand might be similarly concerned about their affected hand’s strength and range of motion. Loss of grip strength and pinch strength is commonly reported following ray resection, particularly following loss of a middle digit [16]. A previous study found that patients who received index ray amputations reported the highest loss of pinch strength of 35.6%, while ray amputations of the middle finger resulted in the highest loss of grip strength of 50.2% [7]. In cases where a patient’s primary concern is their affected hand’s strength and range of motion, proximal phalanx amputation is preferable.

Other considerations of our study showed non-significant differences between patients who received ray and proximal phalanx amputations. Circulation within the affected hand was measured using FLIR and LASCA. Additionally, we did not observe significant changes in health condition, as assessed with the EQ-5D-5L questionnaire.

Limitations of this study include the time-sensitive nature of patient-reported outcomes. As a retrospective study, patient follow-ups occurred at varying time points, which can affect reported results. Additionally, the study was a single-institution analysis. To further these findings, it would be ideal to include additional clinics to encompass a more diverse group of patients, which would improve the translatability of the findings to a larger population. The affected finger was not used to further stratify the data due to the range in our cohort. Future studies examining how the affected finger contributes to functional outcomes of digit amputation would be useful to guide clinicians and patients in amputation-level decision-making. Due to the heterogeneity of the group, it would be useful to include additional factors to evaluate the role of amputation level in functional outcomes. The type of work can greatly differ based on the form of manual labor, and sex differences can play an additional role. Based on these considerations, increased scoring parameters would be useful to gain valuable insight into how lifestyle and work influence patient decision-making regarding finger amputation levels.

Full or partial finger resections present the most common type of upper limb amputation [32]. Due to the prevalent need for these surgeries and the time-sensitive nature of the treatment, it is important for clinicians and patients to make informed decisions regarding amputation levels to maximize postoperative functionality and quality of life. Our study finds ray amputation to be preferable in cases where the patient is concerned about aesthetics and functional outcomes, while proximal phalanx amputation is preferable when the patient is concerned mainly about grip strength and hand dexterity in connection to their work or hobbies. This finding allows for greater personalization of a patient’s treatment to enhance clinical decision-making and for the determination of the amputation level that will provide the best functional outcomes.

## 5. Conclusions

When a patient arrives in a clinic with a traumatic injury to the hand requiring amputation, it is important to make a timely decision on the amputation level that will preserve the highest functionality and postoperative quality of life. Our study contrasts the suitability of ray and proximal phalanx amputations using PRO measures and postoperative functionality tests, including grip strength and blood flow in the affected hand. We found that ray amputation provided more favorable outcomes with regard to quality of life and aesthetics, while proximal phalanx amputation was better suited for individuals concerned about the strength and force of their affected hand. This study is intended to guide clinicians and patients in making informed decisions regarding amputation levels. As personalized medicine becomes increasingly important in ensuring patient well-being, we demonstrated how PROs can be useful for predicting functional outcomes based on patient demographics and needs.

## Figures and Tables

**Table 1:** Patient demographics.

Characteristic	Total (*n* = 40)	Ray Amputation (*n* = 20)	Proximal Phalanx Amputation (*n* = 20)
Age (years)			
Mean	47.5	49.5	45.5
Gender:			
Female	17 (42.5%)	12 (60%)	5 (25%)
Male	23 (57.5%)	8 (40%)	15 (75%)
Hand dominance:			
Dominant	13 (32.5%)	7 (35%)	6 (30%)
Non-dominant	27 (67.5%)	13 (65%)	14 (70%)
Affected finger:			
Digitus 2	17 (42.5%)	8 (40%)	9 (45%)
Digitus 3	6 (15%)	2 (10%)	4 (20%)
Digitus 4	13 (32.5%)	7 (35%)	6 (30%)
Digitus 5	4 (10%)	3 (15%)	1 (5%)

**Table 2:** Summary of patient-reported outcome questionnaries.

	Mean ± SEM		*p*-Value
	Ray Amputation	Proximal Phalanx Amputation	
DASH score	33.88 ± 2.786	50.13 ± 3.625	0.001
PRWE score	10.11 ± 1.772	24.11 ± 4.692	0.0170
EQ-5D-5L score	0.8962 ± 0.0262	0.8804 ± 0.0270	0.6420
EQ-VAS score	82.89 ± 4.297	72.37 ± 3.253	0.0419

**Table 3:** Stratified DASH scoring.

Characteristic	Mean ± SEM								
	Part A			Part B			Part C		
Type of employment	RA	PPA	*p*	RA	PPA	*p*	RA	PPA	*p*
Manual worker	32.78 ± 3.281	46.62 ± 3.327	0.0413	33.28 ± 2.310	36.22 ± 5.028	0.9888	26.95 ± 3.966	46.43 ± 2.203	0.0016
Non-manual worker	27.90 ± 3.828	40.13 ± 6.344	0.2923	39.47 ± 2.016	34.74 ± 3.454	0.9595	25.40 ± 3.031	41.80 ± 4.169	0.0721
Sex									
Female	35.47 ± 2.934	48.68 ± 2.753	0.0038	43.90 ± 1.285	43.95 ± 2.936	0.999	27.39 ± 1.952	50.78 ± 2.657	0.0001
Male	37.58 ± 2.534	47.54 ± 2.384	0.0417	44.33 ± 2.642	44.95 ± 3.223	0.999	50.84 ± 3.311	51.78 ± 2.557	0.999
Affected hand									
Dominant	26.24 ± 2.091	43.47 ± 3.660	0.0442	41.13 ± 3.970	29.74 ± 3.965	0.4584	37.10 ± 5.869	43.47 ± 5.633	0.9052
Non-dominant	32.78 ± 3.281	41.48 ± 3.645	0.4923	31.89 ± 2.787	39.89 ± 3.979	0.5844	28.01 ± 3.890	42.11 ± 3.147	0.0574

Ray amputation (RA), proximal phalanx amputation (PPA), and *p*-value (*p*).

**Table 4:** Functional outcomes.

	Mean ± SEM		*p*-Value
	Ray Amputation	Proximal Phalanx Amputation	
Numeric Pain Rating			
At rest	1.474 ± 0.2212	2.684 ± 0.3672	0.0022
At motion	2.632 ± 0.2883	3.947 ± 0.5269	0.0094
Grip strength (%)	68.09 ± 3.026	89.74 ± 5.213	0.0449
Wrist range of motion (%)	85	100	.
Cold sensitivity (%)	42	75	
Aesthetics (%)			
Excellent	65	30	
Good	32.5	60	
Unsatisfactory	2.5	10	

## Data Availability

The data presented in this study are available on request from the corresponding author. The data are not publicly available due to restrictions in privacy.
